# Evaluation of Chromium-Crosslinked AMPS-HPAM Copolymer Gels: Effects of Key Parameters on Gelation Time and Strength

**DOI:** 10.3390/gels12010087

**Published:** 2026-01-19

**Authors:** Maryam Sharifi Paroushi, Baojun Bai, Thomas P. Schuman, Yin Zhang, Mingzhen Wei

**Affiliations:** 1 Department of Chemical and Biochemical Engineering, Missouri University of Science & Technology, Rolla, MO 65409, USA; msdrc@mst.edu; 2 Department of Earth Sciences and Engineering, Missouri University of Science and Technology, Rolla, MO 65409, USA; 3 Department of Chemistry, Missouri University of Science and Technology, Rolla, MO 65409, USA; tschuman@mst.edu; 4 Department of Petroleum Engineering, College of Engineering and Mines, University of Alaska Fairbanks, Fairbanks, AK 99775, USA; yzhang35@alaska.edu

**Keywords:** AMPS-HPAM gel, chromium crosslinkers, CO_2_ resistance gel, gel strength, gel time, ionic coordination

## Abstract

Controlling CO_2_ channeling in heterogeneous reservoirs remains a major challenge for both enhanced oil recovery (EOR) and secure geological storage. AMPS-HPAM copolymers exhibit high-temperature resistance and brine tolerance compared with conventional HPAM gels, making them well suited for the harsh environments associated with CO_2_ injection. Chromium-based crosslinkers (CrAc and CrCl_3_) were investigated because sulfonic acid groups in AMPS can coordinate with trivalent chromium ions, enabling dual ionic crosslinking and the formation of a robust gel network. While organic crosslinked AMPS-HPAM gels have been widely studied, the behavior of chromium-crosslinked AMPS-containing systems, particularly their gelation kinetics under CO_2_ exposure, remains less explored. This experimental study evaluates the gelation behavior and stability of chromium-crosslinked AMPS-HPAM gels by examining the effects of the polymer concentration, molecular weight, polymer–crosslinker ratio, temperature, pH, salinity, and dissolved CO_2_. The results clarify the crosslinking behavior across a range of formulations and environmental conditions and establish criteria for designing robust gel systems. Gelation times can be controlled from 5 to 10 h, and the resulting gels maintained structural integrity under CO_2_ exposure with less than 3.6% dehydration. Long-term thermal testing has shown that the gel remains stable after 10 months at 100 °C, with evaluation still ongoing. These results demonstrate that chromium-crosslinked AMPS-HPAM gels provide both durability and tunability for diverse subsurface conditions.

## 1. Introduction

Over the past several decades, CO_2_ injection has been widely applied for enhanced oil recovery (EOR); however, its low viscosity and high mobility often lead to preferential flow through high-permeability zones, fractures, and thief zones, reducing the sweep efficiency and increasing operational costs in heterogeneous reservoirs [1,2,3]. These limitations highlight the need for effective conformance control strategies capable of regulating CO_2_ flow within heterogeneous reservoirs.

To address these issues, in situ polymer gels originally developed for water conformance control have been applied for CO_2_ conformance improvement at the field scale since the 1970s [4,5]. These gels are injected as gellant solutions and undergo gelation under reservoir conditions, forming a semi-solid network that reduces permeability in high-flow areas while allowing for continued production from lower-permeability regions [6,7]. This selective plugging redirects CO_2_ toward unswept areas, improving the sweep efficiency and overall recovery in heterogeneous reservoirs [8].

Studies show that among all polymers, hydrolyzed polyacrylamide (HPAM), particularly when crosslinked with chromium, has shown adaptability to a variety of demanding reservoir conditions [9,10]. HPAM is widely used in field applications due to its resistance to bacterial degradation, excellent water solubility, effective mobility control, and cost efficiency [11]. For example, Syed et al. demonstrated that chromium-crosslinked polyacrylamide gels reduced the gas permeability by up to 90% in porous and fractured media, confirming their good sealing performance under high-salinity conditions [4].

However, under CO_2_-rich conditions, the dissolution of CO_2_ into formation brine generates an acidic environment that can accelerate the hydrolysis and chemical degradation of the polymer backbone, ultimately compromising the gel integrity [12,13]. Sun and coworkers reported network collapse and instability in HPAM-based gels under CO_2_ conditions [14], and Seright et al. showed that conventional HPAM-based gels often degrade under high temperatures and salinity [15].

To address these limitations, the chemical modification of HPAM has been widely explored, with the incorporation of functional monomers such as 2-acrylamido-2-methylpropanesulfonic acid (AMPS) shown to substantially improve thermal resistance and salinity tolerance [16,17]. Zhang et al. [18] investigated the effect of the AMPS content on the performance of AM/AMPS copolymer gels with organic crosslinkers under high-temperature, high-salinity conditions. Their results showed that increasing the AMPS content generally improved the thermal stability of the polymer backbone, but beyond an optimal range, excessive AMPS reduced the gel strength and gelation ability, indicating that a balance between stability and mechanical performance is necessary. Gel systems formulated with a polymer containing approximately 25% AMPS exhibited excellent heat resistance, a controllable gelation time, good injectivity, and effective plugging behavior in both laboratory tests and field applications in the Taklamakan Desert, where temperatures reached 130 °C and the salinity was about 71,695 mg/L.

In another study, Guo et al. [19] developed a high-temperature-, high-salinity-resistant polymer gel using an acrylamide AM–AMPS copolymer crosslinked with hydroquinone (HQ) and hexamethylenetetramine (HMTA). The gel exhibited excellent stability at 150 °C in simulated brine with an ultra-high salinity of 2.5 × 10^5^ mg/L. Their results showed that the crosslinker concentration was the dominant factor controlling both the gelation time and long-term stability.

Despite advances in AMPS-modified gels, a challenge remains in achieving reliable control over the gelation kinetics and strength under CO_2_-rich reservoir conditions [20]. Most reported AMPS gel systems rely on organic crosslinkers, which often exhibit relatively slower gelation behavior, increasing the risk of premature plugging or poor placement. In contrast, previous studies have rarely examined chromium-based inorganic crosslinkers in AMPS-containing gel systems, particularly with respect to controllable gelation behavior under CO_2_-rich conditions.

Furthermore, the coupled effects of the polymer chemical composition and crosslinker ratio on the gelation kinetics of inorganic crosslinked systems remain insufficiently understood, particularly under CO_2_-rich conditions where temperature, pH, and salinity strongly influence crosslinking behavior [21].

Although AMPS-modified polymer gels and chromium-crosslinked HPAM systems have been reported separately, the application of chromium-based crosslinkers to AMPS-containing copolymers has received limited attention, and their gelation behavior and stability under CO_2_-relevant conditions remain insufficiently understood.

This study investigates how the formulation parameters, including the polymer concentration, molecular weight, and polymer–crosslinker ratio, interact with environmental conditions such as pH, salinity, temperature, and dissolved CO_2_. These factors directly influence the gelation kinetics and crosslinking efficiency of the resulting gel network.

Rheological experiments were conducted to analyze its viscosity evolution and elasticity. These tests demonstrated that the gel formulation maintains stable rheological properties in supercritical CO_2_, where gel systems commonly experience viscosity loss and network collapse. This combination of a controllable gelation time during injection and minimization of degradation and syneresis in CO_2_-rich environments highlights the potential of the developed gel system for improved CO_2_ conformance control applications.

## 2. Results and Discussion

### 2.1. Polymer Screening

The initial polymer screening focused on identifying a gel system with sufficient CO_2_ resistance, chemical stability, and compatibility with reservoir-relevant conditions. Among the polymers evaluated with different AMPS contents (5, 10, 15, and 20%), AN125 (SNF Floerger, Andrézieux-Bouthéon, France) containing 20% AMPS exhibited the highest resistance to CO_2_ exposure. This polymer showed reduced syneresis and improved stability under acidic conditions, evidenced by minimal viscosity reduction following CO_2_-induced pH changes. The enhanced CO_2_ resistance of AN125 is attributed to the presence of sulfonate groups, which are less susceptible to hydrolysis and maintain strong hydration shells in acidic environments.

After selecting the polymer type, the influence of the polymer concentration on the gel formation behavior was examined to balance network development and practical handling. At polymer concentrations below approximately 4000 ppm, the formulations remained free-flowing, indicating that the polymer chain density was insufficient to support the formation of a continuous crosslinked network. As the concentration increased to around 6000 ppm, stable and self-supporting gels were obtained, reflecting effective polymer–crosslinker interactions and sufficient chain overlap to promote three-dimensional network formation. At polymer concentrations of 10,000 ppm, the system exhibited excessively high viscosity before gelation, suggesting overly dense chain packing that could limit gel placement and propagation.

These observations reflect the progressive increase in the effective crosslinking density with the polymer concentration. At low concentrations, limited chain overlap restricts network connectivity, whereas at excessively high concentrations, dense polymer packing constrains chain mobility and reduces practical applicability. Based on these considerations, a polymer concentration of 5000 ppm was selected for subsequent rheological studies as a compromise between stable gel formation and practical handling.

### 2.2. Crosslinker Screening

This investigation aimed to identify crosslinkers capable of providing controlled gelation behavior suitable for effective placement. Therefore, a range of inorganic crosslinkers, including Ni-Acetate·4H_2_O, NiCl_2_·6H_2_O, CoCl_2_·6H_2_O, ZrCl_4_, ZrAc, AlCl_3_, CrCl_3_, and CrAc, were investigated to examine their influence on the gelation behavior and network stability. Distinct gelation responses were observed across the tested systems. Certain crosslinkers resulted in excessively rapid gelation, limiting injectivity, whereas others produced weak or unstable gels under CO_2_ exposure. In contrast, chromium-based crosslinkers (CrCl_3_ and CrAc) provided a favorable balance between the gelation time and gel strength, forming stable gel networks that maintained integrity under simulated CO_2_ injection conditions. Based on these observations, CrCl_3_ and CrAc were selected for further investigation in subsequent gelation kinetics and rheological studies.

### 2.3. Time-Dependent Gelation Behavior and Rheological Correlation

**Figure 1:**
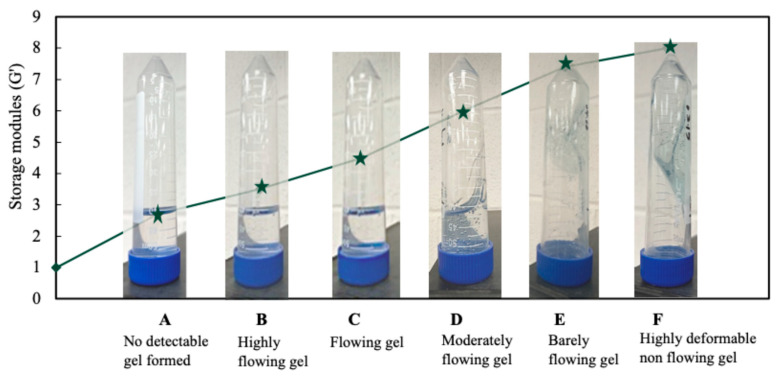
Time-dependent gelation stages (codes A–F) for selected polymer gel formulation (5000 ppm polymer, Cr^3+^-to-polymer ratio of 1:100), with corresponding storage modulus (G′) values obtained from oscillatory rheological measurements.

Figure 1 presents the time-dependent gelation behavior of the selected formulation (5000 ppm polymer, Cr^3+^-to-polymer ratio of 1:100), classified into distinct gelation stages denoted as codes A–F based on the macroscopic flow behavior. To quantitatively define the mechanical significance of these visual classifications, oscillatory rheological measurements were performed for samples corresponding to each gelation code under identical formulation and aging conditions.

The results show a clear correlation between the gelation codes and the measured storage modulus (G′). Samples classified as codes A–B exhibited low G′ values, characteristic of liquid-like behavior with negligible elastic response. At intermediate stages (codes C–D), G′ increased by orders of magnitude, indicating the onset of elastic network formation and partial gel development. At advanced gelation stages (codes E–F), the system displayed high and stable G′ values, reflecting the formation of a fully developed, elastic three-dimensional gel network. The strong correlation between the visual gelation codes and measured G′ values demonstrates that the macroscopic bottle-test classification provides a reliable framework for predicting gel mechanical properties.

### 2.4. Effect of Polymer Molecular Weight and Concentration

#### 2.4.1. Polymer Molecular Weight

The polymer molecular weight is a key parameter influencing the gelation kinetics and network development in chromium-crosslinked polymer systems. Variations in the chain length affect both the availability of reactive functional groups and the extent of chain entanglement, which together govern the rate and efficiency of gel formation. To clarify this influence, polymers with different molecular weights were examined under identical crosslinking conditions.

**Figure 2:**
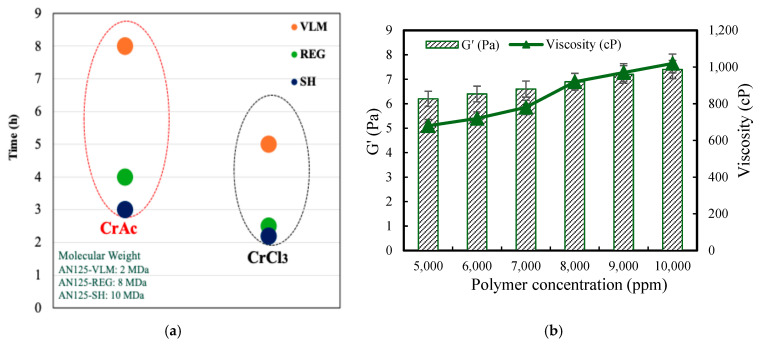
Effects of polymer properties on gelation and rheological behavior: (**a**) gelation time as a function of polymer molecular weight (2 MDa, 8 MDa, and 10 MDa) under identical crosslinking conditions, the red and black dashed circles highlight the grouping of gelation times for different polymer molecular weights when crosslinked with chromium acetate (CrAc) and chromium chloride (CrCl_3_), respectively, and (**b**) variation in viscosity and storage modulus (G′) as a function of polymer concentration, showing increased viscosity and elasticity at higher concentrations.

Figure 2a shows the gelation behaviors of polymers with molecular weights of 2 MDa (VLM), 8 MDa (REG), and 10 MDa (SH) crosslinked with chromium-based crosslinkers. A clear molecular-weight-dependent trend is observed, in which increasing molecular weight leads to progressively faster gel formation. The 10 MDa polymer exhibits the shortest gelation time, followed by the 8 MDa polymer, while the 2 MDa polymer shows significantly delayed gelation.

This trend is attributed to the molecular characteristics of high-molecular-weight polymers. Longer polymer chains provide a greater number of reactive functional groups, such as amine or carboxylic acid moieties, which enhance coordination interactions with chromium-based crosslinkers (CrCl_3_ or CrAc). In addition, higher-molecular-weight polymers exhibit increased chain entanglement, which facilitates rapid network formation. The combined effects of increased crosslinking sites and enhanced chain entanglement accelerate three-dimensional gel network development, leading to a significant reduction in the gelation time compared to lower-molecular-weight polymers.

#### 2.4.2. Polymer Concentration

Beyond molecular-weight effects, the polymer concentration plays an equally important role in defining gel rheological behavior by influencing the chain packing and crosslink density. Changes in concentration directly influence both viscous and elastic responses. To examine this effect, the viscosity and elastic modulus were evaluated over a range of polymer concentrations.

Figure 2b presents the variation in the viscosity and storage modulus (G′) as a function of the polymer concentration. A pronounced increase in both the viscosity and G′ is observed with the increasing polymer concentration, indicating the progressive strengthening of the gel network.

This behavior arises from the increased density of polymer chains at higher concentrations, which intensifies intermolecular interactions and restricts chain mobility, resulting in higher viscosity [22]. Simultaneously, the number of available crosslinking points within the system increases, producing a denser and more interconnected gel network. The higher crosslink density enhances the elastic character of the gel, as reflected by the increased G′ values, enabling more effective energy storage and recovery under deformation [23,24]. As a result, higher polymer concentrations promote the stronger network formation, improved chain entanglement, and enhanced mechanical stability of the gel structure.

### 2.5. Effect of Crosslinker Concentration

The crosslinker concentration affects both the gelation kinetics and mechanical integrity of polymer gels. By controlling the availability of coordination sites and the rate of network formation, variations in the crosslinker concentration directly influence the gelation time, elastic response, and overall gel stability. To clarify these effects, the influence of the chromium chloride (CrCl_3_) and chromium acetate (CrAc) concentrations was examined.

**Figure 3:**
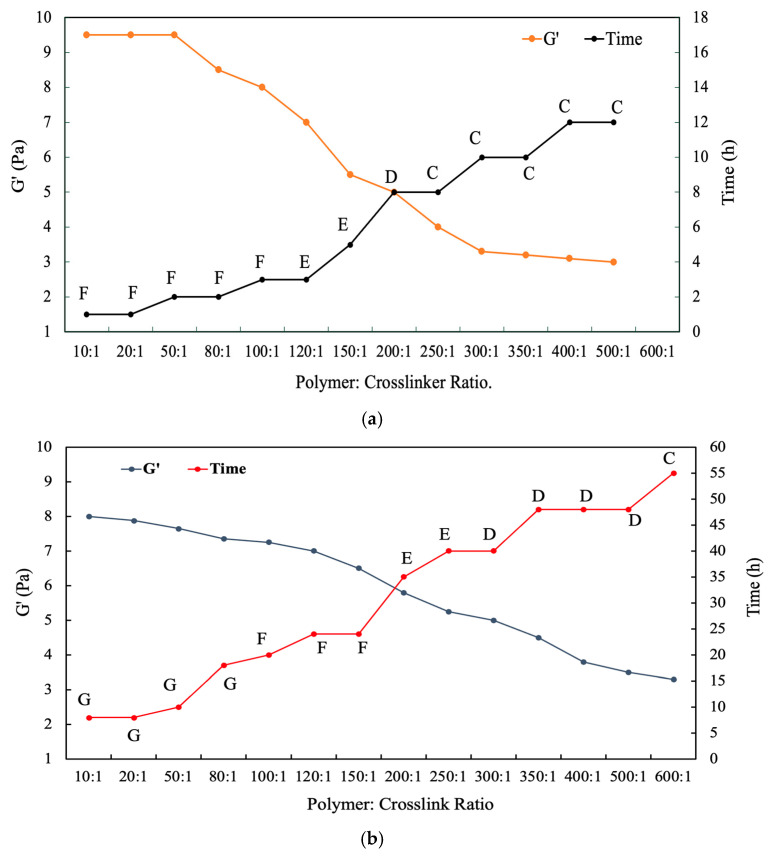
Effect of crosslinker concentration on gelation behavior and mechanical properties of polymer gels crosslinked with (**a**) chromium chloride (CrCl_3_) and (**b**) chromium acetate (CrAc): gelation time and corresponding storage modulus (G′) as function of polymer–crosslinker ratio. Uppercase letters (A–G) represent qualitative gel strength classifications based on the Sydansk gel code, ranging from flowing or weak gels (A–C) to moderately strong and rigid gels (D–G).

Figure 3a,b show the dependence of the gelation time and storage modulus (G′) on the crosslinker concentration for CrCl_3_ and CrAc systems, respectively. For both crosslinkers, the gelation time decreases continuously with the increasing crosslinker concentration. At high polymer–crosslinker ratios (≥300:1), gelation times are relatively long, typically more than 7 h, indicating slow network formation. As the concentration increases, intermediate ratios (100:1–250:1), the gelation time decreases sharply, approximately 3–6 h, reflecting accelerated crosslinking and the faster establishment of the gel network. At lower concentrations (10:1–50:1), the reduction in the gelation time becomes less pronounced, approaching a lower limit of less than 3 h, suggesting a diminishing kinetic benefit once sufficient crosslinking sites are available.

In parallel, the gel strength exhibits a systematic increase with the increasing crosslinker concentration. For both CrCl_3_ and CrAc systems, G′ rises steadily as the crosslinker concentration increases to 8–10 Pa, indicating the progressive stiffening of the gel network. At low concentrations, the gels display relatively low G′ values, corresponding to loosely crosslinked structures with limited elastic response. As the crosslinker concentration increases, G′ increases markedly, reflecting higher crosslink density and enhanced elastic behavior. At the highest concentrations investigated, G′ approaches a plateau, suggesting that the network stiffness becomes limited by the polymer chain availability rather than the crosslinker concentration.

A comparison between the two crosslinkers reveals distinct differences in their gelation behavior. At comparable crosslinker concentrations, CrCl_3_ consistently produces shorter gelation times than CrAc, indicating faster coordination and network formation. However, the corresponding G′ values for CrCl_3_-crosslinked gels remain lower than those obtained with CrAc. In contrast, CrAc yields gels with longer gelation times but higher G′ values across the entire concentration range, demonstrating higher network strength and elasticity.

These observed trends indicate a tradeoff between the gelation rate and mechanical strength that depends on the crosslinker type and concentration. Increasing the crosslinker concentration enhances both the gelation kinetics and gel stiffness, while the choice of crosslinker governs the balance between rapid gel setting and extended gelation time. This tunability enables the crosslinker concentration and type to be selected according to specific CO_2_-EOR operational requirements.

### 2.6. Effect of Temperature in Different Brine Compositions

Temperature strongly affects how fast polymer gels form, especially under saline reservoir conditions [25]. In addition, the ionic composition of the brine can influence crosslinking reactions and the development of the gel network [26]. To examine the combined effects of temperature and the brine composition, the gelation behavior was studied over a range of temperatures using three different brines (B1, B2, and B3) with chromium acetate (CrAc) and chromium chloride (CrCl_3_) as crosslinkers. The polymer concentration (AN125, 5000 ppm) and crosslinker ratio (Cp:Ccr, 50:1) were consistent across all experiments, categorized under Sydansk Code grade C. Brine solutions (B1, B2, and B3) were prepared with controlled ionic strengths. The brine compositions are as follows:

- B1: 2.50 wt% NaCl, 0.06% CaCl_2_, 0.12% MgCl_2_.
- B2: 1.35 wt% NaCl, 0.03% CaCl_2_, 0.06% MgCl_2_.
- B3: 1.25 wt% NaCl, 0.06% CaCl_2_, 0.12% MgCl_2_.

**Figure 4:**
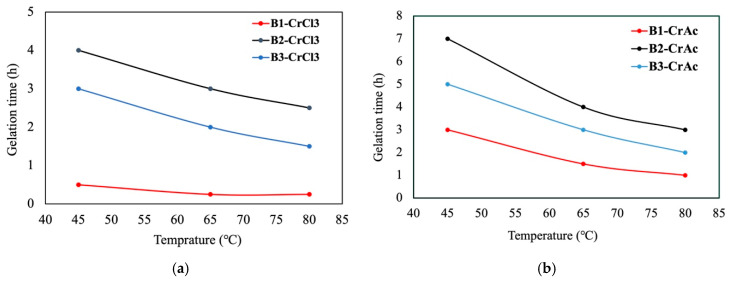
Effect of temperature on gelation time for polymer gels prepared in different brine compositions (B1, B2, and B3), using (**a**) CrCl_3_ and (**b**) CrAc as crosslinker.

Figure 4 illustrates that increasing temperature reduced the gelation time for all brine compositions. This behavior aligns with the expected acceleration of gelation kinetics at higher temperatures, as elevated thermal energy facilitates faster crosslinking reactions. However, distinct differences were observed between the gelation times of the three brine compositions. The gelation time for B2 (7–4 h) was consistently higher than that of B3 (5–3 h), which, in turn, exceeded that of B1 (3–1 h), across the temperature range studied. This ordering suggests that variations in the ionic composition and salinity of the brines significantly influence the gelation process. B2, with a lower concentration of CaCl_2_ and MgCl_2_ compared to B1, exhibited slower gelation, likely due to the reduced availability of divalent ions that contribute to network formation. Conversely, B3, with half the NaCl concentration of B1 but equivalent levels of CaCl_2_ and MgCl_2_, showed an intermediate gelation time. This shows the role of NaCl in modifying the ionic strength and, consequently, the gelation kinetics.

For CrCl_3_, a similar trend was observed, with increasing temperature reducing the gelation times. However, the gelation times were generally shorter than those with CrAc. For CrCl_3_, B3 showed the longest gelation time (4–2.5 h), followed by B2 (3–1.5 h), and B1 exhibited the shortest gelation time (less than 1 h). This pattern highlights the greater influence of the NaCl concentration in CrCl_3_ systems, where higher NaCl levels lead to faster gelation. The presence of divalent ions (Ca^2+^ and Mg^2+^) in B3 and B2 slowed down the gelation process, as these ions tend to form a more rigid crosslinking network, thereby increasing the time required for gel formation. Overall, both crosslinkers exhibit clear temperature sensitivity; however, CrAc provides longer gelation times and higher stability in brines with moderate to high ionic strength, whereas CrCl_3_ enables much faster gelation, particularly in high-NaCl environments. This distinction allows crosslinker selection to be tailored to specific CO_2_-EOR operational requirements.

### 2.7. Effects of Different Salts

To evaluate the polymer gel performance under specific conditions, various salts were used to investigate their influences on the gelation behavior and rheological properties. This study aimed to identify the most effective salts for enhancing the gel strength, stability, and gelation time under controlled experimental conditions.

All experiments were conducted using an AN125 polymer at 8000 ppm, a polymer–crosslinker ratio of 100:1 with chromium acetate (CrAc), and a temperature of 45 °C. The salts screened included sodium chloride (NaCl), potassium chloride (KCl), calcium chloride (CaCl_2_), magnesium chloride (MgCl_2_), sodium sulfate (Na_2_SO_4_), potassium carbonate (K_2_CO_3_), and sodium dihydrogen phosphate (NaH_2_PO_4_), enabling direct comparisons of monovalent and divalent cations (Na^+^, K^+^, Ca^2+^, Mg^2+^) and different anions (Cl^−^, SO_4_^2−^, CO_3_^2−^, H_2_PO_4_^−^).

**Figure 5:**
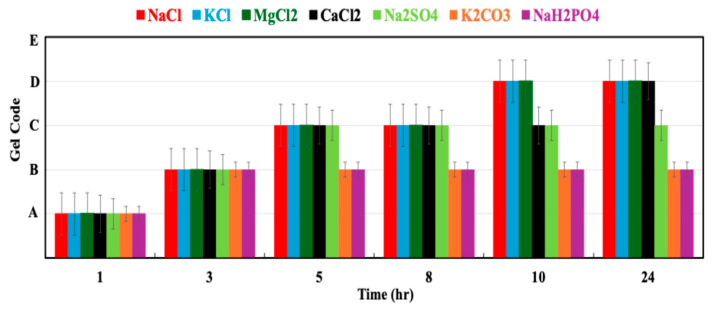
Effect of salt type on gel strength development of polymer gels at different ionic strength levels, showing evolution of storage modulus (G′) over time for gels prepared with monovalent and divalent salts. Error bars represent ± one standard deviation based on three independent measurements.

Figure 5 presents the evolution of the storage modulus (G′) for gels prepared with different salts at comparable ionic strength levels. Systems containing NaCl, KCl, MgCl_2_, and CaCl_2_ consistently formed stable gels with measurable and increasing G′ over time, indicating sustained network development. In contrast, gels prepared with Na_2_SO_4_, K_2_CO_3_, and NaH_2_PO_4_ exhibited lower G′ values and less consistent gel formation, demonstrating reduced network stability under the tested conditions.

Among the chloride-based salts, NaCl and KCl produced nearly identical G′ evolution profiles and comparable final gel strengths (Figure 5), indicating that the type of monovalent cation has a limited effect on the gel strength under these conditions. This similarity is consistent with the comparable ionic size and hydration characteristics of Na^+^ and K^+^, which primarily act to screen electrostatic repulsion between negatively charged polymer chains and facilitate uniform crosslinking [20,27].

Gels prepared with MgCl_2_ exhibited higher G′ values than those formed with monovalent salts at similar ionic strengths (Figure 5), indicating increased network rigidity. This behavior is consistent with the higher charge density of Mg^2+^, which enhances electrostatic interactions with polymer functional groups and increases the effective crosslink density. In comparison, CaCl_2_-based gels showed lower G′ values than MgCl_2_ systems under the same conditions, although stable gel formation was still observed. The reduced gel strength in Ca^2+^-containing systems correlates with the larger ionic radius and lower charge density of Ca^2+^ [28], which weakens electrostatic interactions with the polymer chains and limits the crosslinking efficiency.

Salts containing multivalent anions (Na_2_SO_4_ and K_2_CO_3_) were excluded from further investigation because visible precipitation was observed during gel preparation, leading to disrupted gel structures and unstable rheological behavior. Although NaH_2_PO_4_ induced partial gel formation, the resulting gels exhibited reduced G′ values and poorer stability under elevated-salinity and -temperature conditions and were therefore not considered suitable for continued study.

Overall, the results in Figure 5 and Table 1 demonstrate that the gel performance is strongly dependent on the cation type rather than the ionic strength alone. Based on the gel strength development, stability, and reproducibility, NaCl, KCl, CaCl_2_, and MgCl_2_ were selected for further investigation, while the remaining salts were excluded.

### 2.8. Effect of pH on Gelation Time and Strength

pH is a key parameter influencing both the kinetics of gel formation and the mechanical strength of polymer gels, as it directly affects polymer ionization and crosslinking reactions [29]. Understanding how pH governs gelation behavior is essential for identifying conditions that maximize gel strength while maintaining practical gelation times under reservoir conditions.

**Figure 6:**
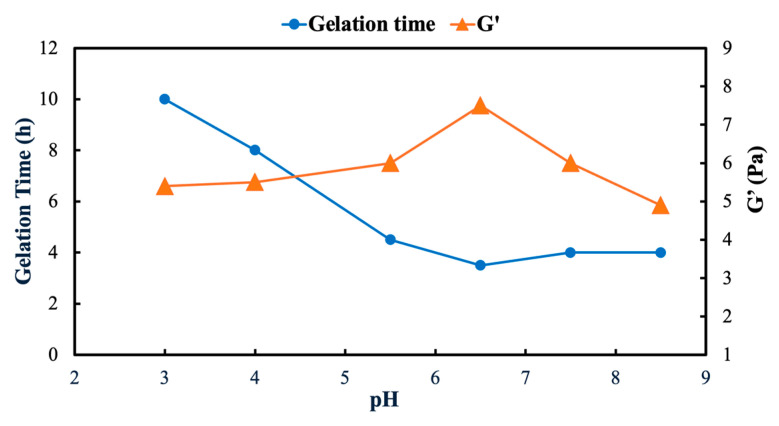
Effect of pH on gelation time and gel strength (storage modulus, G′) of synthesized polymer gel prepared with AN125 polymer (8000 ppm) crosslinked with chromium acetate (CrAc) at 45 °C.

Figure 6 shows the effect of pH on the gelation time and gel strength for the synthesized hydrogel prepared using an AN125 polymer (8000 ppm) crosslinked with chromium acetate (CrAc) at a polymer–crosslinker ratio of 100:1 and a temperature of 45 °C. The initial pH of the polymer solution was adjusted to 8, while the initial pH of the gel was set to 7, ensuring consistent gelation behavior across all experiments.

As shown in Figure 6, the gelation time exhibits a clear dependence on the pH. A minimum gelation time of approximately 4 h is observed at a pH of around 6.5, indicating the most rapid gel formation under the investigated conditions. As the pH shifts toward either more acidic or more alkaline values, the gelation time progressively increases, reaching approximately 10 h, which reflects a slower gelation process. This trend indicates the existence of an optimal pH window in which polymer–crosslinker coordination is most favorable.

Gel strength shows a complementary trend. As the pH increases toward 6.5, the gel strength rises steadily and reaches a maximum value of approximately 8 Pa. Beyond this point, further increases in the pH result in a gradual decline in the gel strength to approximately 6 Pa. This indicates the presence of an optimal pH window wherein the gel network achieves maximum mechanical integrity.

These trends reflect the strong influence of pH on polymer crosslinker interactions and network structures. At intermediate pH values, functional groups along the polymer chains are optimally ionized, promoting effective coordination with Cr^3+^ ions and resulting in a well-developed three-dimensional network. At lower pH values, excessive protonation reduces the availability of coordination sites, while at higher pH values, changes in polymer conformation and possible competitive interactions limit the crosslinking efficiency. As a result, both the gelation kinetics and gel strength are maximized within a specific pH range rather than under extreme pH conditions.

### 2.9. Stability Tests of the Gel

Evaluating the resistance of polymer gels to CO_2_ exposure and elevated temperatures is essential for evaluating their application for the CO_2_-EOR projects. Under reservoir conditions, gels are subjected to supercritical CO_2_ environments and prolonged thermal stress, which can induce chemical degradation, syneresis, or structural collapse. Therefore, the stability of the synthesized gel was examined through CO_2_ resistance and thermal stability tests.

#### 2.9.1. CO_2_ Resistance

The CO_2_ resistance test was conducted under controlled conditions to simulate high-pressure CO_2_ injection environments. The gel sample was exposed to a pressure of 1150 psi and a temperature of 45 °C for 24 h.

**Figure 7:**
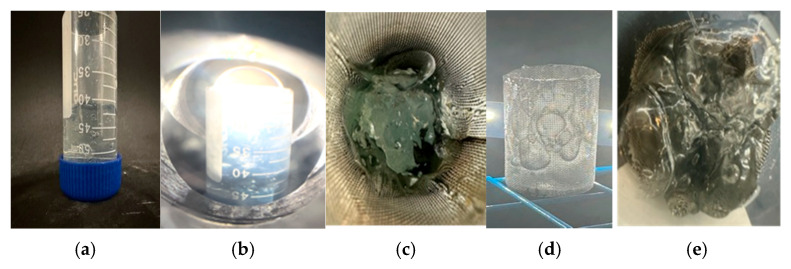
CO_2_ resistance test equipment and gel (**a**–**c**) before and (**d**,**e**) after CO_2_ exposure at 1150 psi and 45 °C for 24 h.

Figure 7 shows the CO_2_ resistance test equipment and gel before and after CO_2_ exposure. Before gas injection, the initial weight of the sample was recorded at 17.27 g (M1). After the test, the sample weight decreased slightly to 16.64 g (M2). The mass loss percentage was determined from the difference between the initial and final sample mass relative to the initial mass and was calculated to be approximately 3.6%. The limited mass loss observed indicates that the gel retained most of its structure and hydration under the tested CO_2_ conditions, demonstrating good resistance to CO_2_-induced degradation.

The CO_2_ resistance is primarily attributed to the AMPS-containing polymer backbone, which introduces sulfonate (–SO_3_^−^) functional groups into the network [18]. Unlike amide groups in conventional HPAM, sulfonate groups are highly stable under acidic conditions and are resistant to hydrolysis induced by carbonic acid formed during CO_2_ dissolution. In addition, the strong hydration shell surrounding the sulfonate groups helps preserve polymer chain expansion and prevents network collapse in the presence of supercritical CO_2_. These characteristics are believed to contribute to the ability of the gel to maintain its three-dimensional structure and resist chemical degradation during CO_2_ exposure.

#### 2.9.2. Thermal Stability Evaluation

**Figure 8:**
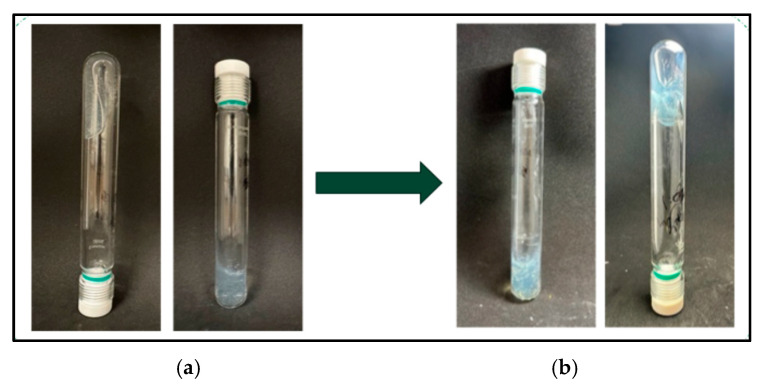
Thermal stability of polymer gel (**a**) before and (**b**) after long-term aging at 100 °C for 10 months.

Figure 8 presents the appearance of the gel before and after thermal aging at 100 °C for 10 months. Throughout the testing period, the gel maintained its structure and stability with no visually observable degradation under the applied thermal-aging conditions. The thermal stability of the gel is again closely linked to the presence of AMPS units within the polymer network. The sulfonate groups in AMPS possess high thermal stability and maintain their ionic character at elevated temperatures, reducing polymer backbone scission and suppressing temperature-induced hydrolysis. Furthermore, coordination bonding between Cr^3+^ ions and the polymer chains is stabilized by the strong electrostatic interactions associated with sulfonate groups, allowing the crosslinked network to remain intact during prolonged thermal exposure. As a result, the gel exhibits sustained mechanical integrity even after long-term aging at 100 °C. Overall, the combined CO_2_ resistance and thermal stability results demonstrate that incorporating AMPS into the polymer structure enhances gel durability. The hydrolysis-resistant sulfonate functionality and stable coordination interactions enable the gel to withstand acidic CO_2_ environments.

From a field application perspective, the observed gelation times on the order of several hours allow sufficient time for gel placement and injectivity before in situ gel formation for CO_2_-EOR conformance control treatments. The moderate elastic modulus values and tunable gelation behavior achieved through adjustment of the polymer concentration, crosslinker type, temperature, and brine composition suggest that the system can be adapted to reservoir-specific operating conditions. These laboratory-scale trends provide practical guidance for formulation screening prior to core flooding and field-scale evaluation.

## 3. Conclusions

This study evaluated the gelation behavior, stability, and CO_2_ resistance of an AMPS-containing polymer gel under conditions relevant to CO_2_-EOR and CO_2_ storage applications. The results indicate that controlled chromium-based crosslinking enables a balance between gel strength and injectivity, with the elastic modulus (G′) increasing to approximately 10 Pa while the gelation time decreases from about 10 h to less than 3 h as the crosslinker concentration increases, depending on the crosslinker type.

The gel exhibited a stable performance over a broad pH range from 3 to 9, with the maximum gel strength observed near a pH of 6.5. Outside this optimum, the gel retained sufficient mechanical integrity, indicating tolerance to pH changes associated with CO_2_ dissolution and carbonic acid formation in injection zones. This behavior is consistent with the presence of AMPS sulfonate groups, which are less susceptible to protonation and help preserve polymer–crosslinker coordination under acidic conditions.

CO_2_ resistance testing provided quantitative evidence of the structural stability under high-pressure CO_2_ exposure. After 24 h at 1150 psi and 45 °C, the gel exhibited a mass loss of approximately 3%, corresponding to retention of more than 97% of the initial gel mass. This result indicates limited CO_2_-induced degradation or dissolution under the tested conditions. Quantitative reporting of such CO_2_ resistance metrics remains limited in experimental studies of CO_2_-responsive polymer gels.

Long-term thermal-aging tests further showed that the gel maintained its structure during continuous exposure at 100 °C for more than 10 months. This observation suggests that the gel network remains stable under prolonged thermal stress within the investigated conditions.

Overall, the results suggest that incorporation of AMPS functionality combined with controlled chromium crosslinking can enhance gel stability in CO_2_-rich, acidic, saline, and elevated-temperature environments. Within the scope of the laboratory conditions studied, the gel exhibits tunable gelation behavior, CO_2_ resistance, and thermal stability that are relevant for conformance control applications in CO_2_-EOR and CO_2_ storage operations.

It should be noted that the findings of this study are based on laboratory-scale experiments conducted under controlled conditions. The results are therefore applicable to polymer gels formulated with chromium-based crosslinkers and AMPS-containing polymers within the investigated ranges of temperature, pH, salinity, and ionic composition. Extrapolation beyond these conditions, including alternative polymer chemistries, crosslinking mechanisms, or field-scale reservoir heterogeneity, should be approached with caution. Further investigation is required to investigate the long-term performance and transport behavior under dynamic reservoir conditions.

## 4. Materials and Methods

### 4.1. Materials

The materials used in this study included polymers, crosslinkers, and brine solutions with specific ionic compositions. The AN125 polymer (SNF Floerger, Andrézieux-Bouthéon, France) used in this study is an HPAM-based copolymer containing approximately 20% AMPS. According to supplier specifications, the polymer has a molecular weight of 8 MDa. To evaluate the effect of the molecular weight on the gelation behavior, additional HPAM-based polymer grades with different molecular-weight ranges, low (2 MDa), medium (8 MDa), and high (10 MDa), were also examined as provided by the supplier. The molecular-weight values reported in this study are based on manufacturer data.

The crosslinking agents used were chromium acetate (CrAc) and chromium chloride (CrCl_3_), obtained from Sigma-Aldrich (St. Louis, MO, USA). All brine solutions were prepared using analyticalgrade sodium chloride (NaCl), calcium chloride (CaCl_2_), magnesium chloride (MgCl_2_), and potassium chloride (KCl), also sourced from Sigma-Aldrich. Deionized (DI) water was used to prepare all solutions to eliminate variability due to impurities.

### 4.2. Polymer Preparation and Gel Synthesis

To prepare the polymer solution, AN125 was dissolved in DI water at a concentration of 5000 ppm. The solution was stirred continuously at 300 rpm for 24 h to ensure complete dissolution. The crosslinker solutions were prepared separately by dissolving the appropriate amounts of CrAc and CrCl_3_ in DI water. The polymer and crosslinker solutions were then mixed under controlled conditions to initiate gelation. Gelation tests were conducted at 45 °C in sealed glass vials to simulate reservoir conditions. The polymer–crosslinker mixtures were incubated in a temperature-controlled oven to monitor the gelation time and final gel strength.

### 4.3. Evaluation Methods

The experimental conditions used in this study were selected based on preliminary laboratory screening and reservoir-specific characteristics of Alaska heavy-oil formations. To investigate the gelation behavior, experiments were designed using an AN125 polymer, with an initial screening of the polymer concentration to balance the injectivity and gel strength. A concentration of 5000 ppm was therefore selected for the base formulation, and unless otherwise stated, all experiments were conducted using this polymer concentration, with a crosslinker–polymer ratio (Cp:Ccr) of 100:1. The brine salinity was fixed at 27,500 ppm to replicate the composition of formation water from Alaska heavy-oil reservoirs. A comprehensive series of tests was then conducted to evaluate the effects of the temperature (45–80 °C), crosslinker type (CrCl_3_ and CrAc), polymer molecular weight, polymer concentration, and pH on the gelation behavior and stability under reservoir-relevant CO_2_-EOR conditions. Gelation was conducted at 45 °C to ensure controlled placement and uniform network formation, while subsequent testing at elevated temperatures (80 °C and 100 °C) was performed to evaluate the post-gelation thermal and CO_2_ stability under reservoir-relevant conditions.

**Figure 9:**
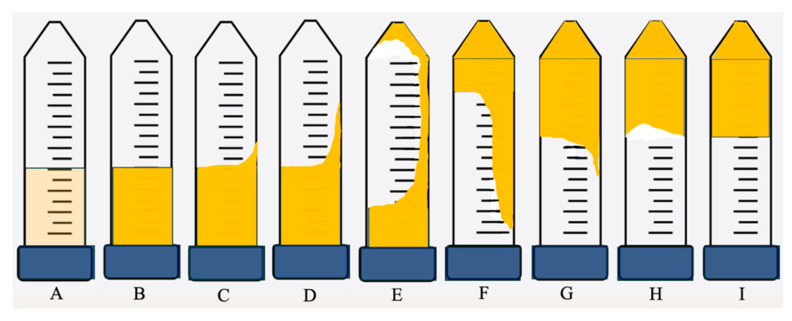
Sydansk gel strength code (**A**–**I**) for visual assessment of gelation state.

Finally, the thermal and CO_2_ stability of the gels was evaluated to simulate harsh reservoir conditions. Rheological properties, including the viscosity and elastic modulus, were evaluated using a rotational rheometer equipped with a parallel-plate geometry, operated in oscillatory mode at ambient temperature, with a constant strain of 1% and an angular frequency of 1 rad s^−1^. Along with rheology experiments, the gels were visually inspected. The gelation time was evaluated using the Sydansk gel strength classification, with Grade C used as a reference state for comparing the gelation behavior across different formulations, as shown in Figure 9 and described in Table 2. The salinity composition consisted of 2.50 wt% NaCl, 0.12 wt% MgCl_2_, 0.06 wt% CaCl_2_, and 0.015 wt% KCl. The samples were also subjected to long-term stability tests at a temperature of 100 °C to evaluate their suitability for CO_2_-resistant applications. All experiments were performed in triplicate unless otherwise stated, and the reported values represent the mean of three independent measurements. The error bars shown in the figures correspond to the standard deviation of the measurements. While minor variability was observed between replicates, the overall trends discussed here were reproducible and consistent across independent measurements.

### 4.4. Experimental Apparatus for Supercritical CO_2_

**Figure 10:**
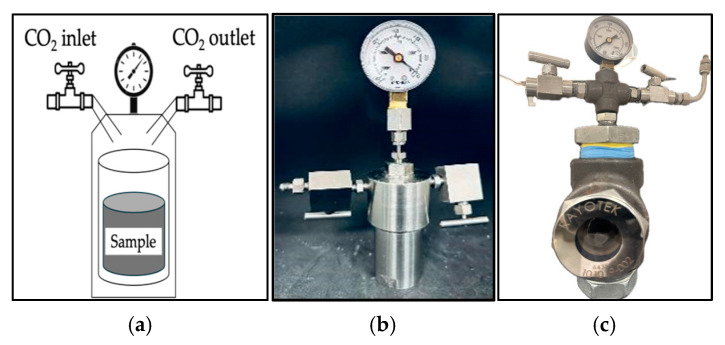
CO_2_ experimental setup used to simulate supercritical conditions: (**a**) schematic diagram of high-pressure CO_2_ vessel system, (**b**) CO_2_ stainless-steel high-pressure vessel, and (**c**) transparent high-pressure vessel made of stainless steel, pressure gauge, and CO_2_ inlet/outlet ports, used for visual observation during testing.

A custom-designed experimental setup was used to simulate supercritical CO_2_ conditions. Figure 10 shows the high-pressure vessel made of a stainless-steel container. The apparatus includes a stainless-steel container equipped with a pressure gauge for monitoring internal pressure and inlet/outlet valves to facilitate CO_2_ injection. Once the CO_2_ is introduced, the container is placed in a temperature-controlled oven to maintain the conditions for achieving the supercritical state. Two variations of the container were utilized: one for withstanding high-pressure environments to measure the dehydration, and a high-pressure transparent model to enable direct visual observation during testing. This configuration ensures precise control and monitoring during the test, which is important for replicating reservoir-like environments in the laboratory setting.

### 4.5. CO_2_ Exposure and Dehydration Measurement

Gel samples were placed in the high-pressure vessel and exposed to CO_2_ at a pressure above the critical pressure of CO_2_ (≥7.38 MPa) to ensure supercritical conditions. The vessel temperature was controlled at the target test temperature (45 °C), and the CO_2_ phase was confirmed based on pressure–temperature conditions relative to the CO_2_ phase diagram. Samples were maintained under CO_2_ exposure for a defined duration of 24 h, during which visual observations were recorded at regular intervals.

Gel dehydration was quantified using a mass-based method by measuring the sample weight before and after CO_2_ exposure. The percentage of dehydration was calculated as the relative mass loss with respect to the initial gel mass. This approach enabled consistent comparison of the gel stability under CO_2_-rich conditions.

## Figures and Tables

**Table 1:** Salt selection and salt effects on gelation and stability.

Ion Type	Salt	Effect on Gelation	Reason for Selection
Monovalent Cations	NaCl, KCl	Enhances gelation by reducing electrostatic repulsion between polymer chains	NaCl prioritized due to wide availability and lower cost
Divalent Cations	CaCl_2_, MgCl_2_	Contributes to gel stability by forming stronger ionic interactions with polymer chains	Higher solubility and effectiveness under experimental conditions
Exclusion (Multivalent Anions)	Na_2_SO_4_, K_2_CO_3_	Precipitation tendencies in presence of divalent cations; negatively impacts gel formation	Excluded due to precipitation and instability in gel formation
Exclusion (Moderate Gelation)	NaH_2_PO_4_	Provides moderate gelation but is less stable under high-salinity and -temperature conditions	Excluded due to limited stability and practical applicability

**Table 2:** Classification and codes for each step (Sydansk Code [25]).

Code	Shape
A	No detectable gel formed
B	Highly flowing gel
C	Flowing gel
D	Moderately flowing gel
E	Barely flowing gel
F	Highly deformable nonflowing gel
G	Moderately deformable nonflowing gel
H	Slightly deformable nonflowing gel
I	Rigid gel
J	Ringing rigid gel

## Data Availability

The data that support the findings of this study are available from the corresponding author upon reasonable request.
